# Management of *Staphylococcus aureus* Bloodstream Infections

**DOI:** 10.3389/fmed.2020.616524

**Published:** 2021-03-05

**Authors:** Aurelia Kimmig, Stefan Hagel, Sebastian Weis, Christina Bahrs, Bettina Löffler, Mathias W. Pletz

**Affiliations:** ^1^Institute for Infectious Diseases and Infection Control, Jena University Hospital, Friedrich-Schiller-University Jena, Jena, Germany; ^2^Department of Anesthesiology and Intensive Care Medicine, Jena University Hospital, Friedrich-Schiller-University Jena, Jena, Germany; ^3^Center for Sepsis Control and Care, Jena University Hospital, Friedrich-Schiller-University Jena, Jena, Germany; ^4^Division of Infectious Diseases and Tropical Medicine, Department of Medicine I, Medical University of Vienna, Vienna, Austria; ^5^Institute of Medical Microbiology, Jena University Hospital, Friedrich-Schiller-University Jena, Jena, Germany

**Keywords:** *Staphylococcus aureus* blood stream infections, endocarditis, diagnostic and therapeutic algorithm, current recommendations, open questions

## Abstract

*Staphylococcus aureus* bloodstream infections are associated with a high morbidity and mortality. Nevertheless, significance of a positive blood culture with this pathogen is often underestimated or findings are misinterpreted as contamination, which can result in inadequate diagnostic and therapeutic consequences. We here review and discuss current diagnostic and therapeutic key elements and open questions for the management of *Staphylococcus aureus* bloodstream infections.

## Introduction

*Staphylococcus aureus* (*S. aureus*) is one of the leading pathogens causing community-acquired and hospital-acquired bloodstream infections ranking second after *Escherichia coli*. Incidences were estimated between 10 to 30 cases per 100,000 person-years (1) and hospital mortality is high, ranging between 15 and 40% (2, 3).

The gram-positive pathogen has developed several strategies to adapt to the infected host by evading the hosts immune system, e.g., it can form biofilms, adhering to intravascular catheters and implantable medical devices (4). Furthermore, intracellular persistence in different kind of host cells such as epithelial and endothelial cells or osteoblasts, has been described (5). Inside these biofilms and host cells, *S. aureus* can form slow growing subpopulation, so called small colony variants (SCV). These colonies display a lower metabolic activity and have an increased tolerance against antibiotics, which can result in refractory or chronic infections and relapses (6, 7).

In 8–15% of the patients, hematogenous spread may also lead to later secondary complications such as endocarditis, vertebral osteomyelitis, abscesses, and implant associated infections of prosthetic joints, electronic cardiac devices etc., which can occur up to weeks or months after the primary infection. Notably, patients with community-acquired SA-BSI and patients with prolonged bacteremia have an increased risk for secondary foci (8). Further risk factors for complications are inadequate antibiotic treatment, an unknown primary focus of infection or insufficient source control (9).

Given the high rates of mortality and morbidity associated with SA-BSI the management differs from bloodstream infections with other bacteria. A structured management in diagnostic and treatment is crucial for an optimal outcome. Several studies have shown that an adherence to treatment guidelines and infectious disease bedside-consultation can lead to a reduction of mortality by up to 50% (10–12).

## Current Diagnostic and Treatment Standards

### Diagnostic Key Principles

As a principle, blood cultures positive for *S. aureus* always need to be respected as a clinically significant finding and should result in an appropriate treatment. Blood culture contamination with *S. aureus* is a very rare event (<5%) and due to the high mortality and the high risk of serious complications associated with *S. aureus* bloodstream infections (SA-BSI), a prompt therapy is generally recommended (12–14).

*S. aureus* detection in urine culture (*S. aureus* bacteriuria) should lead to the search of an underlying bloodstream infection as *S. aureus* rarely causes genuine urinary tract infection, but is most likely filtrated through the kidneys (15, 16). An exception are patients with urinary tract foreign bodies and/or after urological interventions. In these patients, the urinary tract can be the primary focus of a bloodstream infection with the pathogen. *S. aureus* bacteriuria in patients with a SA-BSI has been associated with a worse outcome (17). Careful patient history and thorough physical examination with a special emphasis on potential foci are mandatory. Most frequent sources of SA-BSI are intravascular catheters and soft tissue infections (18). Further diagnostics have to be performed depending on clinical findings. In up to one third of all cases, however, septic embolism remains inapparent in the clinical examination and will be diagnosed solely in an extended diagnostic work-up imaging (19).

In order to prevent further spreading of *S. aureus* causing secondary septic metastases, source control must be carried out as quickly as possible. Infected foreign bodies incl. vascular catheters or cardiac electronic devices have to be removed quickly and completely in addition to an adequate antibiotic treatment (20, 21). If vascular catheters have been *in situ* during bacteremia, a removal should be considered even if another site is suspected as focus of the SA-BSI since catheters remain the most frequent primary source of infection and moreover there is a high risk of secondary catheter colonization (22–25). Endocarditis occurs in about 10–20% of patients with SA-BSI and worsens the patient's prognosis (26, 27). The diagnostic sensitivity of transesophageal echocardiography (TEE) is twice as high as the one of a transthoracic echocardiography (TTE) and should thus be used preferably (28). Ideally, TEE is performed within a few days (3–5 days) after diagnosis of a SA-BSI. In patients with persisting clinical suspicion of endocarditis and/or positive follow-up blood cultures, a repeated TEE after about 7 days is recommended (29, 30).

Twenty-four hours (up to 72 h at the latest) after the initiation of therapy, follow-up blood cultures are required to evaluate therapy success (31). Positive blood cultures at this point are associated with the presence of septic metastasis, insufficient source control, and consequently with a poorer outcome and therefore require further investigation (31, 32). It has been recommended to take at least two blood culture pairs at each time of collection as the sensitivity of blood culture depends on the number sampled (33, 34). However, at least for further follow-up blood cultures and particularly in intensive care units patients, this has to be weighed with the aims of “patient blood management” (35).

**Figure 1:**
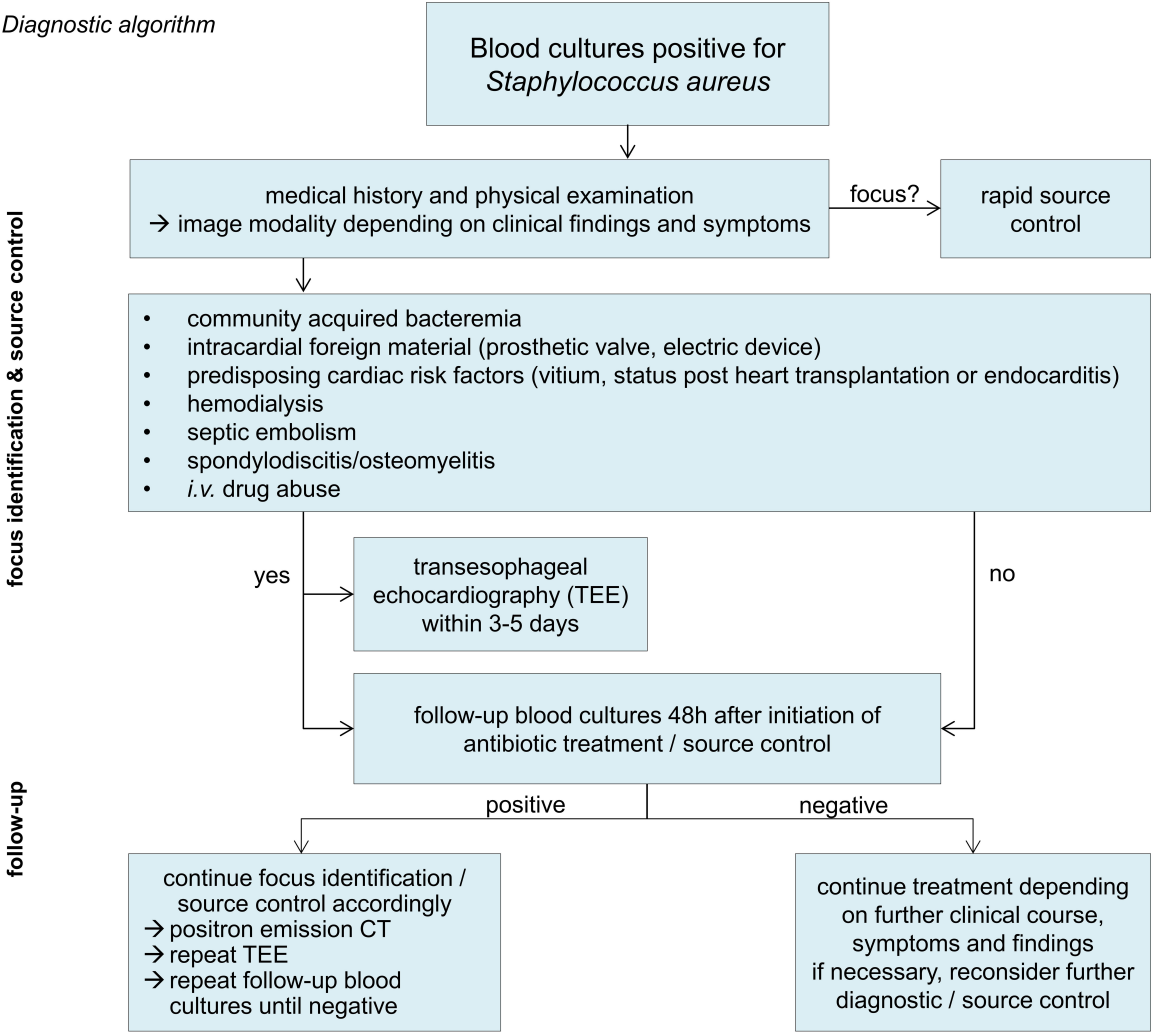
Diagnostic algorithm.

In patients with positive follow-up blood cultures, a fluorodeoxyglucose positron emission computed tomography scan (FDG-PET CT) should be considered. In a cohort study on 115 patients with gram-positive bacteremia (56% with *S. aureus*), PET-CT imaging had a very high sensitivity and specificity and detected twice as many septic embolisms compared to conventional diagnostic methods (19). An overview of the diagnostic key priciples is shown in Figure 1.

### Therapeutic Key Principles

Antibiotics of choice in the therapy of bloodstream infections by methicillin (oxacillin) sensitive *S. aureus* (MSSA) are beta-lactam antibiotics with high activity against *S. aureus*. Best outcomes are being achieved with anti-staphylococcal penicillins (e.g., flucloxacillin) and first generation cephalosporins (cefazolin). A recent meta-analysis showed that cefazolin is not inferior to a therapy with anti-staphylococcal penicillins in the therapy of MSSA bloodstream infections (36). Moreover, cefazolin treatment was associated with a significant lower risk for drug side effects (nephrotoxicity, hepatotoxicity, venous irritation) and was associated with a numerically higher survival rate.

Piperacillin/tazobactam, ceftriaxon, cefuroxim and other broad spectrum beta-lactams should not be used for definite treatment of SA-BSI despite *in vitro* confirmed susceptibility, because they are not only associated with a higher likelihood to select multi-drug resistant pathogens but also with an increased mortality according to retrospective studies (37).

The relevance of penicillin allergy has recently been discussed (38). In case of an IgE-mediated (immediate type) penicillin allergy, daptomycin is recommended as an alternative to β-lactam antibiotics (39). Vancomycin application was associated with increased mortality compared to β-lactam antibiotics and therefore is not recommended for the definite treatment of MSSA bloodstream infections (40).

Antibiotics of choice for treating bloodstream infections due to methicillin (oxacillin) resistant *S. aureus* isolates (MRSA) are vancomycin and daptomycin (41). The reference range for vancomycin trough levels is 15–20 mg/l (12, 42). Lower trough levels have been associated with treatment failure (43). Another reason for treatment failure and poorer outcome is a reduced vancomycin susceptibility (44–46). Vancomycin resistance is defined by a minimum inhibitory concentration (MIC) of ≥16 μg/ml, strains with a MIC of 4–8 μg/ml are so called “vancomycin intermediate susceptible *S. aureus*” (VISA) (47). The term “hetero-resistant” VISA (hVISA) refers to *S. aureus* strains that are primarily within the susceptible range but contain subpopulations which can develop a reduced susceptibility under exposure to vancomycin (48). Patients with hVISA BSI are at higher risk of having a persistent bacteremia, which is associated with higher mortality in SA-BSI (49, 50). Altogether, occurrence of isolates with reduced susceptibility to vancomycin has increased over the last years probably also due to more frequent use of vancomycin in patients with MRSA infections (51). However, prevalence is still low with under 5% and daptomycin remains an effective treatment option in these isolates (52, 53).

Daptomycin monotherapy (with 8–12 mg/kg ideal bodyweight) is considered an equivalent alternative. Daptomycin has been shown to be inactive in patients with pneumonia, probably because it is inactivated by pulmonary surfactant and therefore unsuitable in patients with pneumogenic infection (54). Linezolid should not be used for MRSA bloodstream infection due to its bacteriostatic effect (55).

The new MRSA effective cephalosporins (ceftarolin, ceftobiprole) and lipoglycopeptides (dalbavancin) should not yet be used as first choice as there are no randomized controlled trials in patients with MRSA bloodstream infections (56–58).

**Figure 2:**
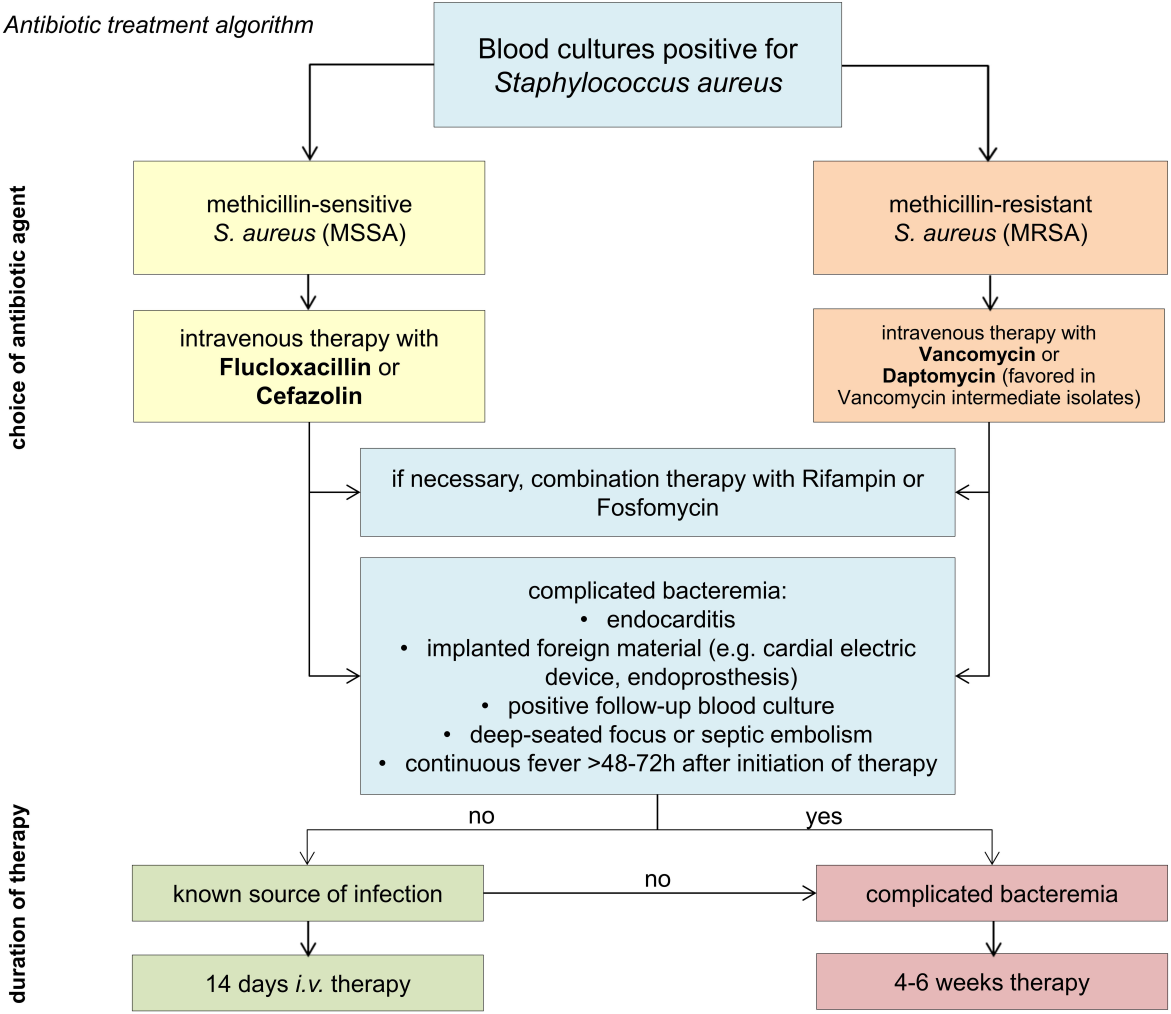
Antibiotic treatment algorithm.

Treatment duration depends on the clinical course and classification of infection as “complicated” or “uncomplicated” SA-BSI (Box 1). For patients with uncomplicated bloodstream infection an intravenous antibiotic treatment is recommended for at least 14 days. SA-BSI classified as complicated or without known source of infection require a minimum of 4–6 weeks of therapy. After completion of at least 14 days i.v. antibiotic therapy, an oral sequential therapy can be considered (Box 2). For an overview of the therapeutic principles (see Figure 2).

Box 1 **“Uncomplicated” *Staphylococcus aureus* bloodstream infection (41).**

no evidence of endocarditis in the physical examination or echocardiography

no implanted foreign bodies *in situ* (e.g., prosthetic valves, cardiac electronic device, prosthetic joints)

negative blood cultures 48–96 h after initiation of therapy

no evidence of deep-seated focus or septic metastases (e.g., vertebral osteomyelitis)

defervescence within 48–72 h after initiation of therapy

Box 2 **Prerequisite criteria for an oral sequential therapy (59).**

adequate reduction of inflammatory parameters

clinically satisfactory response to treatment

no evidence of abscess, insufficient source control or endocarditis

temperature <38.0°C/100.4°F for >48 h

negative follow-up blood cultures

### Pertinent Open Questions

Despite its frequency, the quality of evidence for the treatment of patients with SA-BSI is insufficient with only few randomized controlled trials and only a handful of larger multicenter retrospective studies available (3, 26, 60–62). The following chapter summarizes open questions and associated recent data.

## Current Quality Evidence and Open Questions

### Who Needs Echocardiography?

Endocarditis is a dreaded complication of SA-BSI. Recently published studies show, that indication for an echocardiography should be risk-adjusted as not all patients have the same risk of acquiring an endocarditis (30, 63–65). Currently, a TEE is recommended in the following situations: community-acquired SA-BSI, prolonged bacteremia, history of intravenous drug abuse or dialysis, cardiac risk factors (foreign bodies, valve defects, history of endocarditis, heart transplant), septic embolism, e.g., vertebral osteomyelitis or cerebral embolism (30, 63, 65). Since clinical presentation of symptoms and findings are not specific, scores may be a helpful tool to identify patients who need transesophageal echocardiography. However, so far only retrospective studies have been performed on this topic (30, 65). Moreover, a large number of risk factors for endocarditis have been described in the literature to date (27, 65, 66) and hence, they apply to a large number of patients in daily clinical routine.

### How Long Do We Have to Treat?

It is recommended to treat an uncomplicated SA-BSI for at least 14 days to avoid relapse (67). Among others, a prospective observational cohort study showed that relapse occurred in up to 8 vs. 0% of patients with short-course therapy <14 days (68). In complicated SA-BSI and episodes with unknown primary focus of infection international experts recommend at least 28 days of antibiotic therapy (67). However, underlying evidence is not satisfactory (69) and guidelines and recommended quality indicators are mainly based on retrospective data (62, 70–73). Moreover, recommendations regarding treatment standards mostly refer to studies showing an improvement of outcomes by means of adherence to a bundle of measures or infectious specialist consultations (67). Randomized trials are needed to provide more evidence to what extend respective treatment elements such as duration of antibiotic therapy contribute to an improvement of patient outcomes.

### Can We Switch to Oral Antibiotics?

Currently at least 14 days of parenteral antibiotic therapy are recommended in patients with a SA-BSI. At what point, or whether a switch to oral therapy is safe at all, is a matter of debate. In a recently published randomized multicenter trial by Iversen et al. changing to oral antibiotic combination treatment (e.g., dicloxacillin or linezolid plus rifampcin) was noninferior to continued intravenous treatment for patients with left side endocarditis including patients with *S. aureus* endocarditis (59). However, the outcome of the study was mainly carried by patients with native valve endocarditis due to streptococci (196 out of 400 patients). Therefore, it is unclear whether the conclusion of the study also applies to patients with *S. aureus* endocarditis. Comparable evidence for other foci of SA-BSI is lacking. Obviously, a sufficient oral bioavailability of the applied antibiotic is essential in any case. Possibly, the SABATO trial which finished recruitment recently will provide new aspects (61). The major objective of the randomized, parallel-group, observer-blinded, clinical non-inferiority trial is to demonstrate that in patients with low-risk SA-BSI a switch from intravenous to oral antimicrobial therapy is non-inferior to a conventional course of intravenous therapy.

### What Is the Role of Combination Therapy?

The role of combination therapy, particularly with rifampicin or fosfomycin, in addition to a ß-lactam antibiotic, in MSSA, or vancomycin, in MRSA, SA-BSI, is a matter of debate. Theoretically, combination therapy could lead to a higher bactericidal activity compared to antibiotic monotherapy and synergistic effects could occur. Combination therapy may be superior in the eradication of intracellular staphylococci and biofilms on foreign materials and thus reduce the risk of secondary late infection and recurrence (74, 75).

However, disadvantages of combination therapy, such as drug-related side effects and interactions need to be considered in the benefit-risk assessment. Clinical studies have not shown a benefit of routine combination therapy for all patients with SA-BSI (3, 76–79). The multicenter randomized ARREST trial by Thwaites et al. (3) found no significant effect of additional rifampicin on treatment failure, disease recurrence, or death.

### The Role of Computerized Decision Systems and Phone Consultations

A structured management in diagnostic and treatment is crucial for an optimal outcome. Several studies have shown that an adherence to treatment guidelines and particularly infectious disease bedside-consultation can lead to a reduction of mortality by up to 50% (10–12). Given the limited availability of infectious diseases physicians, who are usually based in larger hospitals and the standardized management outlined above, novel approaches are currently under investigation, such as computerized decision support systems and phone consultations, to improve outcome by providing respective expertise also in smaller hospitals (80, 81). However, it has yet to be proven whether these approaches are as effective as bedside-consultations by infectious diseases physicians.

## Conclusion

Management of patients with SA-BSI remains challenging as mortality and complication rates are high and we still lack sufficient high-quality evidence addressing the most pertinent questions. A structured management preferably provided by an antibiotic stewardship team or infectious consultation including a standardized diagnostic work-up and therapeutic approach is prerequisite for all patients with SA-BSI to improve treatment quality and patient outcomes.

## Author Contributions

AK, SH, SW, CB, and MP: drafting of the manuscript. All authors: critical revision of the manuscript and additional important intellectual content.

## Conflict of Interest

The authors declare that the research was conducted in the absence of any commercial or financial relationships that could be construed as a potential conflict of interest.
